# Update on Pulmonary Ossifications in the Differential Diagnosis of Solitary Pulmonary Nodules

**DOI:** 10.3390/jcm10204795

**Published:** 2021-10-19

**Authors:** Jan F. Gielis, Lawek Berzenji, Vasiliki Siozopoulou, Marloes Luijks, Paul E. Y. Van Schil

**Affiliations:** 1 Department of Thoracic and Vascular Surgery, Antwerp University Hospital, University of Antwerp, 2650 Antwerp, Belgium; lawek.berzenji@uza.be (L.B.); paul.vanschil@uza.be (P.E.Y.V.S.); 2 Department of Pathology, Antwerp University Hospital, 2650 Antwerp, Belgium; vasiliki.siozopoulou@uza.be; 3 Department of Pathology, ZNA Stuivenberg, 2060 Antwerp, Belgium; marloes.luijks@zna.be

**Keywords:** pulmonary ossification, solitary pulmonary nodule, dendriform ossification, nodular ossification

## Abstract

Pulmonary ossifications have often been regarded as rare, post-mortem findings without any clinical significance. We have investigated the occurrence of pulmonary ossifications in patients undergoing thoracic procedures, and how this may affect the differential diagnosis of solitary pulmonary nodules. In addition, we have performed a literature search on the occurrence and possible pathogenesis of these ossifications. From January 2008 until August 2019, we identified pulmonary ossifications in 34 patients who underwent elective pulmonary surgery. Pre-operative imaging was unable to differentiate these ossifications from solid tumors. A definitive diagnosis was made by an experienced pathologist (VS, ML). The PubMed database was researched in December 2019 with the search terms “pulmonary ossifications”; “heterotopic ossifications”; and “solitary pulmonary nodule”. In total, 27 patients were male, with a mean age of 63 ± 12 years (age 41 to 82 on diagnosis). All lesions were identified on thoracic CT and marked for resection by a multidisciplinary team. A total of 17 patients were diagnosed with malignancy concurrent with ossifications. There was a clear predilection for the right lower lobe (12 cases, 35.3%) and most ossifications had a nodular form (70.6%). We could not identify a clear association with any other pathology, either cancerous or non-cancerous in origin. Oncologic or pulmonary comorbidities did not influence patient survival. Pulmonary ossifications are not as seldom as thought and are not just a curiosity finding by pathologists. These formations may be mistaken for a malignant space-occupying lesion, both pre-and perioperatively, as they are indistinguishable in imaging. We propose these ossifications as an underestimated addition to the differential diagnosis of a solitary pulmonary nodule.

## 1. Introduction

Mature bone tissue in the alveolar or interstitial spaces is a remarkable finding that was first described in an obduction report by the anatomist Herbert Von Luschka in 1856 [1]. These pulmonary ossifications (PO) are occasionally found in the lung parenchyma of patients undergoing a thoracotomy for a solitary pulmonary nodule (SPN). The pathogenesis of this phenomenon is not completely elucidated yet. Although PO case series are often reported by pathologists as a curiosity finding post-mortem, we argue that pulmonary ossifications are more commonly observed than often thought, and are clinically relevant to the surgeon, radiologist, and pathologist alike. In a period of 11 years (2008–2019), we have built a case series of 34 patients in which PO were found after a pathological analysis of the resected lung tissue. Pre-operative imaging was not able to differentiate these PO from other causes of an SPN, either malignant or benign. The diagnosis may be overlooked or missed due to insufficient awareness. This paper addresses the need for clinicians to be conscious of PO as a clinical entity that should be included in the differential diagnosis of an SPN.

### 1.1. Morphology

**Figure 1:**
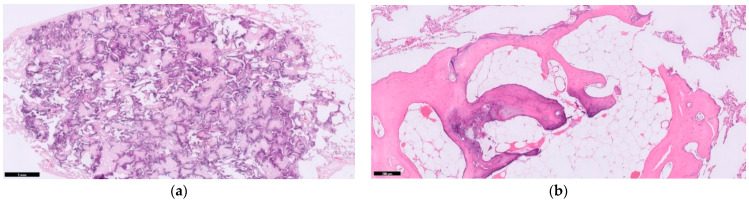
Different forms of pulmonary ossification. (**a**) A rounded nodular ossification; (**b**) Coral-like dendriform ossification, interwoven through the alveolar septa and containing fatty marrow deposits with haematopoetic stem cell tissue (arrow). HE stain, magnification (**a**) 40×; (**b**) 100×.

Ossification is defined as deposits of calcium in a collagen matrix in the presence of osteoblastic cells. Two distinct types of PO are distinguished in literature: nodular PO and dendriform PO. Nodular PO are secluded to the alveolar space and are often round. Dendriform PO branch through the interstitium of the septa in a tree-like coral pattern. Sometimes, the bone tissue has matured to encase osteoblasts, osteoclasts, and bone marrow with fatty cells and haematopoetic stem cell tissue (Figure 1). Even with high-resolution computed tomography, it is difficult to discern the nodular from dendriform PO. Differentiating between these two types clinically has less importance as both types may co-exist in the pulmonary tissue of the same patient [2].

### 1.2. Genetic Predisposition

There is not much indication that genetic anomalies are responsible for the development of PO, although there has been an occasional finding of familial clustering in a father and son [3]. Deletions in the TT7CA gene, important in intestinal development, result in immunodeficiency, repeated infections, and pneumonitis. In one case, this was associated with dendriform PO [4]. However, there is only anecdotal evidence that a genetic predisposition may facilitate the formation of PO and further investigations of familial links are required.

### 1.3. Pathogenesis

In literature, dendriform ossifications are associated with chronic lung injury and inflammation and nodular ossifications are more often found in patients with passive congestion due to chronic heart failure. This may explain why PO are more often found in the lower lobes, as blood accumulates there in patients with insufficient cardiac function. The subsequent extravasation and degradation of red blood cells result in hemosiderin deposits. These react with calcium that is released by injured tissue to form precipitating salts. The process is further propagated by phospholipids released from injured cell walls that degrade into fatty acids which may also bind free calcium. This ultimately results in the deposition of a calcified matrix in or around the alveoli. Long term lung injury appears to cause metaplasia of fibroblasts into osteoblasts. Two important enzymes activated by lung injury and inducing osteoblast activity are alkaline phosphatase (AP) and transforming growth factor-beta (TGF-β). AP is an enzyme that is abundantly present on type II pneumocytes and catalyzes the production of free phosphate which increases the calcium-phosphate product [2]. TGF-β further stimulates collagen II production by fibroblasts and is extensively involved in the pathogenesis of lung fibrosis [5]. Macrophages may differentiate into osteoclast-like multinucleated giant cells in the presence of macrophage colony-stimulating factor (M-CSF) and interleukin-4, thus inducing osteogenesis and bone turnover [6]. The generation of bone marrow in this process is not clearly understood.

## 2. Materials and Methods

We conducted a retrospective study to identify any cases of PO at the Antwerp University Hospital, a tertiary care center in Belgium. From January 2008 until August 2019, pathologic specimens from all patients undergoing thoracic surgery were examined for the presence of pulmonary ossifications. We collaborated closely with our center’s pathologists dedicated to thoracic pathology (VS, ML) to identify any PO and determine whether their growth pattern could be considered as either nodular (NPO), or dendriform (DPO), or both. All specimens were formalin-fixed and stained with hematoxylin-eosin for subsequent light microscopy. Nodular calcifications without any evidence of ossifications (i.e., presence of either osteoblasts, osteoclasts, or osseous matrix) were omitted from this series. All ossifications lesions were previously identified on thoracic computed tomography (CT) and considered for resection by a multidisciplinary oncologic team. 

A literature search was conducted through the PubMed database in December 2019 with the search terms “pulmonary ossifications”; “heterotopic ossifications”; and “solitary pulmonary nodule”.

## 3. Results

A total of 34 cases were identified from the period 2008–2019, with an average incidence of 1.1% to a total of 3122 thoracotomies or thoracoscopies performed at our center during this period. This equated to three cases per year.

**Figure 2:**
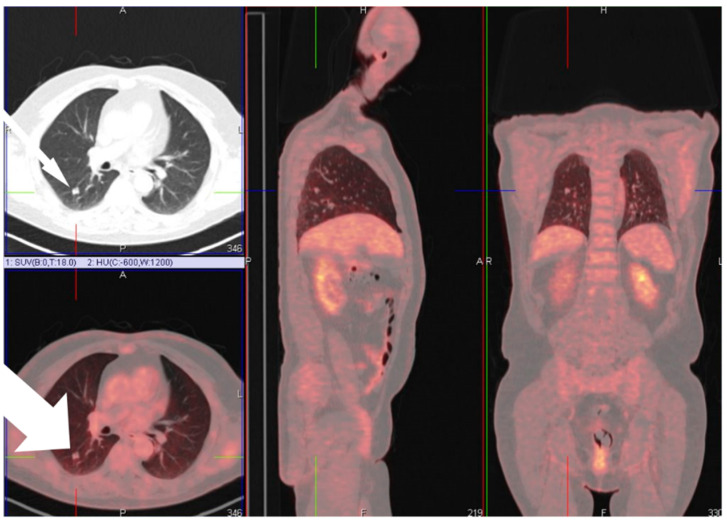
PET-CT showing moderate FDG-avidity of a lesion on PET scan in the right lower lobe with evidence of calcification or ossification on the diagnostic CT. FDG, fluorodeoxyglucose.

Most patients were male (28 patients, 82.4%) with a mean age of 63 years upon diagnosis (range 41 to 82). Nine patients (26.5%) had a history of obstructive lung diseases such as asthma or chronic obstructive pulmonary disease (COPD), and only three patients (8.8%) suffered from interstitial lung fibrotic disease. Two patients (5.88%) were diagnosed with congestive heart failure. Suspected malignancy was, in all cases, the reason to perform surgery. This oncologic diagnosis was confirmed in 19 patients (55.9% of cases). We could not demonstrate any correlation between the imaging and operative findings. Due to their small size, not all pathologically proven ossifications could be visualized on chest CT scans. A representative image is presented in Figure 2, where a positron emission tomography combined with computed tomography (PET-CT) scan showed a moderate FDG uptake with clear evidence of calcification or ossification in the lesion on the CT alone.

We observed a clear predilection for the lower lobes (left lower lobe in 12 cases, or 35.3%, and right lower lobe in eight cases, or 23.5%), and most ossifications were found in the right lung (21 cases, 61.7%) (Figure 2). We identified 24 cases of nodular ossification (70.6%). In six patients (17.6%), bone marrow formation was observed, equally divided between the nodular and dendriform ossifications.

A Pearson correlation test was performed to determine correlations between parameters. We observed moderate correlations (ρ = 0.417; *p* = 0.013) between the presence of bone marrow and arterial hypertension, and elder age (ρ = 0.352; *p* = 0.038). There were no significant correlations between the location of the tumor and the presence of marrow or the difference between nodular or dendriform PO.

**Figure 3:**
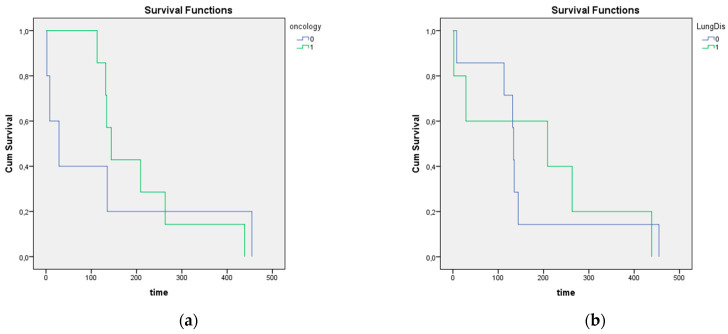
Kaplan–Meier survival analysis of survival time in patients suffering from oncologic disease (**a**) or pulmonary disease (**b**). We did not find any significant differences in survival time.

A Kaplan–Meier analysis with Log rank test was performed to determine whether there were any differences in survival time between patients where PO were found either with and without malignancy, or with and without any pulmonary comorbidity (Figure 3). The survival distributions for these factors were both statistically not significantly different with a *p*-value of 0.719 and 0.760, respectively. Mean survival time (MST) for oncologic patients was 204.7 weeks, 95% confidence interval (CI) 118.9–290.6 weeks; for non-oncologic patients MST was 125.8 weeks, 95% CI 0–293.6 weeks; for patients with pulmonary disease MST was 158.3 weeks, 95% CI 17.0–299.5 weeks; for patients without pulmonary disease 185.4 weeks, 95% CI 17.0–299.5 weeks.

## 4. Discussion

In this paper, we describe 34 cases in total of PO, confirmed after a histopathological analysis, in a retrospective case series that spans eleven years. Originally defined as a post-mortem diagnosis and a rather seldom finding, we argue that PO are truly more common than often thought and deserve to be admitted to the list of possible differential diagnoses for a solitary pulmonary nodule. More awareness of this entity by radiologists, pulmonologists, surgeons, and pathologists may lead to a better understanding of PO and, possibly, an increased prevalence in pre- and postoperative reports.

We found that PO most often occur in elder patients and mostly in men. Although the past years have seen an increase in papers reporting on PO, the diagnosis is seldom made following surgery, and most papers focus on post-mortem observations of this condition [7]. Final pathological examinations often mention the presence of calcification but omit incidental findings of osteoblast activity and bone marrow formation. It takes a dedicated and aware pathologist to accurately describe this condition. With an increasing sensitivity of high-resolution CT scans in bone window images (width 2500 HU; level 500 HU), radiological findings may help in the differential diagnosis of this entity. The final confirmation, however, still follows after a careful pathological examination. CT was not able to differentiate between the nodular and dendriform types, the latter being only found in 5.6% of ossifications after the pathological analysis, compared to 29.4% in our study [8].

**Figure 4:**
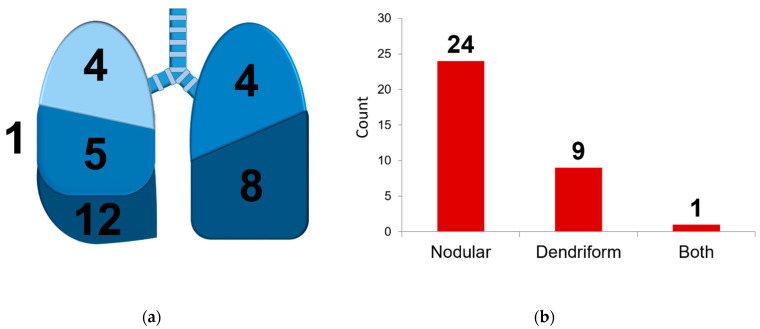
Anatomical and histological characteristics of PO described in this research article. Panel (**a**) shows the anatomical location in the different pulmonary lobes, with one ossification found in the subpleural space. Panel (**b**) shows the differentiation between nodular and dendriform ossification. One specimen contained both nodular and dendriform ossifications.

Differentiating between nodular and dendriform types of PO seems a bit arbitrary. We could not find any clear association with a clinical disease, of which congestive heart failure and pulmonary fibrosis are most commonly found to be associated with PO in literature. The presence of PO does not influence patient survival when suffering from any lung disease or oncologic disease, as demonstrated through the calculated survival functions in Figure 4. Because we found both types of PO to be present in the lungs of the same patient [9], there is probably a significant overlap between both types of PO. The nodular form, usually linked with passive congestive processes, equally appears in patients suffering from chronic obstructive pulmonary disorders in our case series, which are, in the literature, usually associated with dendriform PO. We argue that chronic injury leading to fibrosis and metaplasia, whatever the primary cause, may lead to PO formation.

Furthermore, we confirmed that the nodular form is more abundantly present. When considering the differential diagnosis, dendriform PO may especially be confused with disorders associated with considerable morbidity and mortality such as bronchiectasis, interstitial fibrosis, and even lymphangitic metastasis [7].

We could not find any association between the presence of bone marrow tissue and the differentiation between either NPO or DPO, although the literature suggests that DPO are more commonly associated with marrow formation [7]. The mechanism behind bone marrow formation in PO is not elucidated at all, although there is evidence that the influx of marrow cellular components ensues bone formation during intramembranous ossification [10]. Elder age appears, at least in our study, to favor bone marrow formation. This may imply that marrow formation appears only later on in the process of ossification.

A predilection for the lower lobes was found in our study group, which is confirmed by earlier reports [11]. The base of the lung has a lower ventilation/perfusion ratio compared to the upper lobes, with increased acidity (lower pH because of increased paCO2 at the lung base) and relatively higher blood pressure [12]. These observations may be counter-intuitive: calcification occurs more spontaneously at increased pH, as these circumstances favor AP activity. The upper lung regions are indeed a more alkaline environment. These observations might imply that there is a difference between the pathophysiological mechanisms behind ossification and calcification, and that inflammatory processes, perfusion abnormalities, and increases in shear stress are more important in the development of PO compared to ventilation effects. Anoxia and inflammation may initiate free radical cascades in an acidic environment and promote fibroblastic proliferation. Rising levels of TNF-β, IL-4, and AP during inflammation further induce fibroblast metaplasia into osteoblastic activity [5].

Although PO are sometimes considered a post-mortem rarity, their clinical implications are clear. We advocate for an increased awareness of this curious finding, from the pre-operative diagnosis by a radiologist who may order a high-resolution CT to differentiate PO from other possibly more severe pathologies such as a metastasis, to the thoracic surgeon who suddenly feels an unexpected nodule during thoracotomy, and finally the pathologist encountering bone formation where least expected. With a population that continues to grow older with both cardiac and pulmonary chronic diseases, we can expect an increase in these incidental findings.

## 5. Conclusions

To conclude, we reported a case series of 34 patients with different forms of pulmonary ossification. Although the importance of the antemortem diagnosis of PO is underscored in literature, there is certainly a need for clinicians to be aware of this finding. We did not observe any specific correlated pathologies, either cardiac or pulmonary, with any form of ossification. With an increasing population of elderly patients combatting chronic diseases, we may certainly expect a further and steady increase in the diagnosis of these curious findings. Alertness of clinicians in different aspects of medicine to include PO in the differential diagnosis of pulmonary ossifications may result in a further augmentation in diagnoses. Therefore, we propose PO as a valuable addition to the list of differential diagnoses of an SPN.
